# β1 and β4 integrins: from breast development to clinical practice

**DOI:** 10.1186/s13058-014-0459-x

**Published:** 2014-10-30

**Authors:** Paola Nisticò, Francesca Di Modugno, Sheila Spada, Mina J Bissell

**Affiliations:** 1 Laboratory of Immunology, Regina Elena National Cancer Institute, Via Elio Chianesi 53, Rome, 00144 Italy; 2 Department of Molecular Medicine, Sapienza University of Rome, Via Regina Elena 291, Rome, 00161 Italy; 3 Division of Life Sciences, E. O. Lawrence Berkeley National Laboratory, University of California, Berkeley, 94720 CA USA

## Abstract

Following a highly dynamic and complex dialogue between the epithelium and the surrounding microenvironment, the mammary gland develops into a branching structure during puberty, buds during pregnancy, forms intricate polar acini during lactation and, once the babies are weaned, remodels and involutes. At every stage of menstrual and pregnancy cycles, interactions between the cells and the extracellular matrix (ECM) and homotypic and heterotypic cell–cell interactions give rise to the architecture and function of the gland at that junction. These orchestrated programs would not be possible without the important role of the ECM receptors, integrins being the prime examples. The ECM–integrin axis regulates many crucial cellular functions including survival, migration and quiescence; the imbalance in any of these processes could contribute to oncogenesis. In this review we spotlight the involvement of two prominent integrin subunits, β1 and β4 integrins, in cross-talk with tyrosine kinase receptors, and we discuss the roles of these integrin subunits in the biology of normal breast differentiation and as potential prognostic and therapeutic targets in breast cancer.

**Electronic supplementary material:**

The online version of this article (doi:10.1186/s13058-014-0459-x) contains supplementary material, which is available to authorized users.

## Introduction

Despite progress in the clinical treatment of breast cancer and the availability of new potential drug candidates, the disease continues to progress in part due to a lack of mechanistic understanding of the normal physiology, the mechanisms involved in cancer progression and resistance to therapy [1].

Disorganization of tissue architecture is a characteristic of breast neoplasia and involves an active remodeling of cell–cell and cell–extracellular matrix (ECM) interactions. Integrins, as befits their anointed name in 1992 [2], sense and integrate cues from the ECM to the cytoskeleton, promoting intracellular signals and generating cellular and wide-ranging universal responses, such as proliferation, functional differentiation, survival, polarity and migration. In parallel with the outside-in signaling, integrins respond to intracellular signals and can mediate cell adhesion to the ECM, transmitting forces that may derive either from outside the cell or from intracellular contractility [2],[3]. Integrin cross-talk with growth factor receptors affects the receptor function and many aspects of tumor progression [4]. Accordingly, the expression of integrins is altered in tumors and cancer cells [5] and is associated with poor prognosis, disease progression [6]-[8] and resistance to therapy [9]-[14].

Here we focus on two integrin subunits, β1 and β4 integrins, fundamental in directing polarity and breast tissue structure. In breast epithelium, β1 forms heterodimers with α1, α2, α3, α5 and α6 chains, whereas β4 partners only with α6 [2],[15]. We shall discuss the role of β1 and β4 integrin subunits – with no discussion of the role of their alpha partners – in organization/disorganization of mammary architecture during development, tissue homeostasis, cancer progression and resistance to therapy.

## β1 and β4 integrins are important regulators of mammary gland function and development

**Figure 1:**
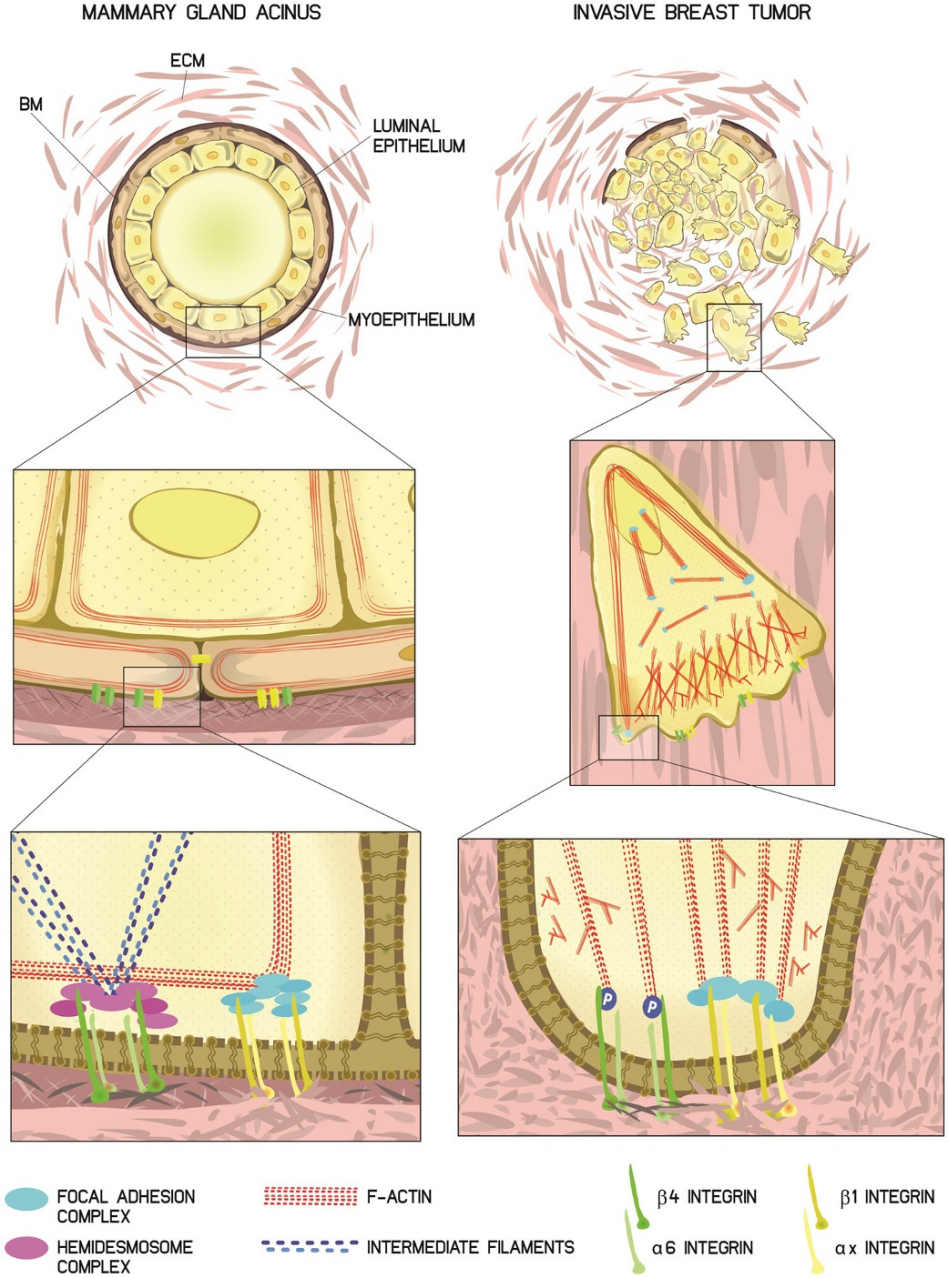
**β1 and** **β4 integrins in normal mammary gland acini and in invasive breast tumors. (Left)** Mammary gland acini consist of a polarized architecture with a pseudo-stratified epithelium, including a luminal epithelium and a myoepithelial layer covered by a basement membrane (BM). Epithelial cells display apico-basal polarity, a cobblestone-like morphology with cortical actin filaments and an apical lumen. In the myoepithelial cells, α6β4 integrin links the cytoskeleton intermediate filaments to the BM through the assembly of hemidesmosomes. β1 integrin heterodimers connect the extracellular matrix (ECM) components and their biochemical and physical cues to the actin cytoskeleton through the focal adhesion complex. **(Right)** Invasive breast tumors lose their organized architecture, bilayered epithelium and BM by upregulating matrix metalloproteinases. Tumor cells lack polarity, change cell shape and display actin protrusions, which mediate cell migration and invasion. α6β4 integrin is phosphorylated (P) following microenvironmental cues and relocates into an F-actin-rich protrusion after hemidesmosome disassembly. β1 heterodimers are localized in invasive protrusions assembled with focal adhesions.

The mammary gland stands out as a unique organ thanks to its remarkable architectural evolution that occurs after its initial fetal development. Remodeling occurs during the major developmental stages of puberty, pregnancy and lactation and every month, driven by the cyclical influence of the reproductive hormones. The development of the organ begins during embryogenesis with a small rudiment referred to as anlage; in males the mammary gland remains undeveloped, but in females it branches into an arboreal – and later a glandular – structure, entailing a complex network of interaction and communication orchestrated by the ECM and the stromal microenvironment. One of the first demonstrations of the importance of integrin signaling in functional differentiation was the involvement of β1 in milk production in a three-dimensional collagen assay [16]. But both β1 and β4 are involved throughout mammary morphogenesis [17]. These two integrins show a similar expression pattern, although they are involved in different adhesion complexes. Upon binding to the appropriate ECM ligand, β1 undergoes conformational changes and its short cytoplasmic tail recruits several cytoplasmic proteins that oligomerize into focal adhesion structures to connect extracellular components, such as fibronectin (FN), collagen and laminins, with signaling components of the cytosol and intracellular actin cytoskeleton [15] (Figure 1, left panel).

The physiological function of β4 in normal breast epithelia is to connect the basement membrane (BM) laminins to the intermediate filament network of the myoepithelial cells through the assembly of adhesive structures – the hemidesmosomes [18] (Figure 1, left panel).

The human mammary gland at the 14th week consists of two layers: an inner layer of central primary bud cells and an outer layer of basal primary bud cells surrounded by the mesenchyme. At 21 weeks, mammary projections sprout from the primary bud into the mesenchyme, the secondary bud stage [19]. At this stage, the basal cells are precursors of myoepithelial lineage, and although alpha-smooth muscle actin-negative, they express α6, β1 and β4 integrins at their plasma membrane [20].

Constant duplication of the primary ducts generates a tree-like pattern spreading throughout the entire fat pad. This branching morphogenesis is an orchestrated process that depends on the ECM, ECM receptors such as integrins, and ECM-degrading enzymes such as matrix metalloproteinases (MMPs), cytokines – like transforming growth factor beta (TGFβ) [21] – and a number of growth factors.

There are conflicting results in culture assays and *in vivo* data on the roles of β1 and β4 in mammary branching, according to the ECM composition and the model used. Notwithstanding this uncertainty, proper branching morphogenesis hinges on the spatial and temporal expression of α2β1 integrin, and its major ligands: types I and IV collagen and laminin, all of which peak during puberty and pregnancy, as shown in a mouse model [22]. The organization of collagen fibers is affected by ECM proteins such as FN [23], and FN fibrils accumulate at the cleft between new buds and are required for branching to occur. Moreover, thick fibrils of collagen around the terminal end buds are deposited in response to exogenous TGFβ [23] and the inhibitory effect of TGFβ signaling on the epithelial branching and ductal growth is crucial for the maintenance of proper ductal spacing, characteristic of the open architecture of the mammary gland [21].

Recently, the role of β1 and ECM degradation in MMP activity was reported by Mori and coworkers, who have shown that MMP14 is highly expressed at the tips of invading mammary end buds during branching in virgin mice. This process requires the reciprocal association of MMP14 and β1, with mitogen-activated protein kinase (MAPK) signaling modulation [24].

In contrast, α6β4 integrin is not essential for proper branching morphogenesis *in vivo*, although in mice lacking α6β4 integrin the rapid advancement of end buds generates a fragile glandular epithelium due to deficiencies in cell–BM adhesion, exacerbated in tissue areas of shear stress [25]. On the other hand, in immortalized normal breast cell lines such as MCF10A, α6β4 integrin is determinant in the formation of hemidesmosomes, and their disruption impedes branching morphogenesis [26].

The functional unit of the mammary gland is the acinus, blind-ended ductules embedded in ECM-enriched, intralobular stroma that develops from the lateral branches of the multibranched mammary tree. The recreation of mammary gland acini in culture has led to an understanding of the different mammary cell functions.

An organotypic acinar structure resembling *in vivo* terminal ductal lobular units has been generated using the breast epithelial cell line HMT-3522-S1 cultured in laminin-rich ECM gels [27]. The HMT-3522-S1 cells form acini with basal–apical polarity when cultured on laminin-111 polymer, which resembles the BM. Integrins β1, β4 and α6 are basally localized [5] and allow proper signaling to establish apical polarity. Integrin α6β4 is critical for the basal polarity in three-dimensional cultured HMT-3522-S1 cells and alteration of this integrin signaling leads to the formation of disorganized colonies and the loss of polarity. Furthermore, the involvement of laminin ligated α6β4 in the formation of mature hemidesmosomes and nuclear factor-κB activation is basic to BM-directed tissue polarity and resistance to apoptosis of polarized mammary structures [28]. Recently, at the molecular level, β1 and the proximal signaling component integrin-linked kinase (ILK) have been shown to control internal protein trafficking and microtubule spatial orientation, required for apico-basal polarity and lumen formation of murine mammary epithelial cells [29]. In mammary epithelial cells cultured on the BM matrix, the deletion of β1 disrupts focal adhesion and impedes acini formation with a reduction of tyrosine phosphorylation in focal adhesion kinase (FAK) and paxillin [30].

During pregnancy and lactation, differentiated structures – alveoli – develop throughout the ductal network to produce milk. β1 is essential in the alveogenesis process, as demonstrated by deleting the integrin in the mouse mammary gland, leading to defects in alveolar development, milk production and integrity of epithelial structures. β1-null alveolar cells were unable to differentiate in the presence of prolactin or to phosphorylate the STAT5 transcription factor, essential for epithelial cell differentiation [30]. Among β1 integrin effectors that control cell differentiation, ILK and FAK have been reported to be located within the same adhesion complex; conditional depletion of ILK, but not of FAK, abrogates differentiation of luminal epithelial cells and lactogenesis *in vivo*[31]. Considering the role of β1 in the control of milk production and secretion, the inhibition of β1–ECM interactions interrupts the signaling that induces β-casein expression [32]. Furthermore, α3β1 integrin is necessary for oxytocin-induced myoepithelial contraction that enables the ejection of milk from the alveolar lumen into the ducts [33].

In summary, mammary branching morphogenesis, acinar polarity, alveogenesis and lactation require a coordinated signaling between the ECM, hormones and growth factors, which are integrated by β1 and β4.

## β1 and β4 integrins in breast cancer

The breast architecture consists of ducts and acini with luminal epithelium lining the ducts and basal myoepithelial cells that rest on the BM and communicate with the ECM via integrins. Great interest is currently focused on understanding the prodigious architecture of the mammary gland and how the vast network of signals, fundamental in sustaining this architecture, is integrated to regulate the normal organ and becomes disrupted in cancer.

Breast cancer is a heterogeneous disease that develops through many stages; the tumor is initiated in the epithelial compartment, although the cause of initiation may be elsewhere. Once abnormal epithelial cells are formed, they pile up and progress into *in situ* and invasive carcinomas, some of which progress to become metastatic. In hyperplasias and *in situ* carcinomas, proliferating epithelial cells lose polarity and bilayered organization, but remain contained within the ductal structures, separated from the breast stroma by myoepithelial cells and BM. Indeed, the breakdown of the myoepithelial cell layer and loss of the BM is peculiar to invasive breast cancer (Figure 1, right panel). An early dysregulation of cell adhesion and ECM pathways is evidenced by differential integrin expression among normal breast, simple hyperplasia, atypical hyperplasia, ductal carcinoma *in situ* (DCIS) and invasive tumors [34]. In human hyperplasias and in benign tumors, integrin expression is predominantly found in myoepithelial cells rather than in luminal cells, reminiscent of the pattern of normal breast. However, in carcinomas the pattern changes to one in which the balance of different subunits is lost [34]. In a small cohort of patients, a higher percentage of β1 expression was found in recurrent DCIS with respect to nonrecurrent cases, suggesting that β1 could be a marker of an aggressive form of DCIS [7]. In patients with invasive breast cancer, high β1 integrin expression was found to be associated with significantly shorter overall and disease-free survival [35],[36].

## What role do β1 and β4 play in tumor formation and progression?

We have taken advantage of a three-dimensional progression model of human breast cancer where the cells became malignant with passage without addition of carcinogens or exogenous oncogenes after removal of epidermal growth factor [27]. The use of this model has led to the identification of structures, key molecules and mechanical cues able to maintain the function and architecture of the mammary gland in culture [37]. Premalignant HMT-3522-S2 cells, derived from normal HMT-3522-S1 cells, provide evidence that breast cells lose architectural integrity before they become malignant. Furthermore, the tumorigenic and invasive counterpart HMT-3522-T4-2 cells are unable to organize and form disordered colonies that continue to grow [27]. β1 and β4 are abnormally expressed and localized in the tumorigenic HMT-3522-T4-2 cells [28]. In these cells the β1-integrin function-blocking antibody, which restores the β1 level present in normal tissue, caused a reversion to a normal phenotype with functionally polarized acinus-like structures, and this occurs only in three-dimensional cultures [5],[13],[27],[37].

The polarized reverted HMT-3522-T4-2 structures had basally organized β4 and contained increased levels of mature hemidesmosomes [28]. On the other hand, transfecting normal HMT-3522-S1 cells with β4 mutant, deleted in the cytoplasmatic tail, in competition with the endogeneous wild-type β4 integrin, disrupts the hemidesmosome formation and perturbs the cytoskeleton organization and BM-directed tissue polarity [28].

## β1 and β4 integrins in breast tumor formation

The role of β1 and β4 in breast tumorigenesis has been shown clearly in both human three-dimensional cultures and mouse models. Polyomavirus middle T transgenic mice express higher β1 in disordered structures than in normal acini at 10 weeks old. Ablation of β1 in the mammary epithelium severely impaired breast tumor formation and β1-integrin-deficient mice show a dramatic reduction in the number of hyperplastic mammary lesions. β1-integrin-null cells had greatly decreased FAK tyrosine phosphorylation and stopped growing [38]. On the other hand, the targeted disruption of FAK function in the mammary epithelium impairs mammary tumor development, and FAK-deficient cells form hyperplastic lesions with a reduced proliferative potential [39].

In mouse mammary tumor virus (MMTV)/ErbB2 mice, conditioned deletion of β1 in mammary epithelial cells resulted in a significant 33-day delay in the induction of mammary tumors and an increase in the number of apoptotic cells, indicating the importance of β1 in mammary tumorigenesis [40]. Recent effort has thus been directed to the dissection of the role of integrins and the associated signaling pathways involved in mammary tumors. Downstream from α3β1 activation, the FAK, Rac1/PAK1, MAPK and c-Jun N-terminal kinase pathways promote prosurvival and proproliferative signals required for the malignant growth of mammary epithelial cells [41]. Moreover, ILK expression has an impact on ErbB2-driven mammary tumor onset, and disruption of ILK inhibits proliferation and invasion and sensitizes ErbB2 tumor cells to apoptotic cell death [42].

Integrin α6β4 was shown to be involved in ErbB2-driven cell proliferation and invasiveness in breast cancer cell lines [43], and in Neu-β4-WT mice the β4–ErbB2 complex enhances activation of STAT3, leading to deregulation of epithelial adhesion, loss of cell polarity, activation of c-Jun and hyperproliferative activity [44]. Deletion of the β4 signaling domain in these mice suppressed the ErbB2-driven mammary gland tumorigenesis [44].

## β1 and β4 integrins in breast tumor progression

Integrins are involved also in progression of the malignant phenotype. For example, epithelial–mesenchymal transition (EMT) – characterized by the loss of cell–cell junction, apico-basal polarity and acquisition of migratory and invasive behavior [45] – involves the alteration of integrin expression and actin cytoskeleton remodeling. EMT is regulated by ECM components as well as soluble growth factors, with TGFβ being the major player [45]. TGFβ regulates expression of integrins, in particular β1, and TGFβ-induced EMT in mammary epithelial cells is mediated by p38 MAPK and β1-integrin activation, shown by blocking β1 integrin and/or inhibiting p38 MAPK [46].

Murine 4 T1 cells are invasive mammary cells that are unable to invade in response to TGFβ when β1 expression is depleted, indicating the necessity of β1 during TGFβ-mediated breast cancer cell invasiveness. β1-deficient 4 T1 cells, stimulated with TGFβ, show an actin cytoskeleton architecture with epithelial features, compared with the elongated morphologies and stress fibers of the parental cells that were treated with TGFβ. On the other hand, β3 integrin expression is necessary in this model for the EMT program [47].

Exogenous addition of TGFβ to nonmalignant MCF10A, overexpressing ErbB2, reorganizes the actin cytoskeleton leading to Rac1 activity, formation of lamellipodia and increased motility. This requires multiple signaling pathways such as phosphatidylinositol-3-kinase (PI3K) and MAPK and TGFβ-stimulated cell migration is prevented by specific inhibition of β1 [48].

To invade, breast cancer cells change their shape and form invadopodia, actin-based invasive protrusions that facilitate the invasion of tumor cells across the BM. β1 function and its cross-talk with epidermal growth factor receptor (EGFR) are crucial for the formation of invadopodia in the MDA-MB-231 breast cancer cell line cultured in both two-dimensional and three-dimensional ECM [49]. Recently, the interaction of metastatic tumor cells with their surrounding ECM was shown to be mediated by filopodium-like protrusions containing β1 integrin, with formation of these structures under control of cytoskeleton-regulatory proteins such as ILK/β-parvin and cofillin [50]. Cytoskeletal configuration and actin stress fibers formation are also peculiar to transition from cellular dormancy to a metastatic proliferative state. β1 activation by FN and/or type I collagen can lead to cytoskeletal organization via the FAK, SRC and myosin light-chain kinase pathways, culminating in the transition from quiescence to proliferation and metastatic growth *in vitro* and *in vivo*[51]. Dormancy has been reported to be suppressed by the cross-talk between α5β1 and the urokinase receptor, which activates extracellular signal-regulated kinase in the presence of FN fibrils [52].

In mouse fibroblasts, the actin regulator MENA interacts with α5β1 integrin and contributes to key α5β1 functions, such as FN fibrillogenesis, cell spreading, motility and activation of adhesion-mediated signaling, such as FAK and paxillin phosphorylation [53]. Actin regulatory proteins thus not only integrate signals from the ECM to the actin cytoskeleton, but are also involved in integrin signaling [53]. Of note, human MENA is overexpressed in human breast cancer, and its splicing was shown to be related to an EMT phenotype [54]. EMT features characterize the claudin-low subtype of breast cancer, and β1 is highly expressed in this subgroup [8]. In a cohort of patients with invasive breast cancer, when β1 is coexpressed with FN, it is associated with decreased overall and disease-free survival [35]. Finally, early establishment of experimental brain metastases, from breast cancer and other organs, was found to be dependent on adhesion to neurovascular ECM. This vessel co-option, and subsequent micro-metastatic perivascular growth, was attenuated in glioblastoma with inhibitory anti-β1 monoclonal antibodies and by knocking out β1 [55].

β4 overexpression was found in basal-like breast cancers, significantly correlating with aggressiveness. A β4 signature, which contains actin binding proteins able to form and stabilize actin protrusion that mediate cell migration and invasion, has been generated [6]. Recently, cross-talk between P-cadherin and the α6β4 signaling pathway has been established and the properties of P-cadherin in inducing stem cell and invasive behavior in basal-like breast cancer cell lines has been ascribed to its cooperation with α6β4 [56]. β4 re-localization to the leading edge of the cells and its association with F-actin to promote cell migration and invasion is induced by epidermal growth factor-mediated phosphorylation, which in parallel liberates β4 from the hemidesmosome structures [57] (Figure 1, right panel).

## β1 and β4 integrins in resistance to therapy

Cross-talk between receptor tyrosine kinase (RTK) and integrins in breast cancer progression is a crucial event and has to be taken into consideration when designing specific breast cancer therapies.

The coupling and bidirectionality of β1 and EGFR signaling has been clearly shown in the organotypic model of breast tumor progression described above [27]. In the HMT-3522-T4-2 cells, EGFR and β1 are upregulated, and inhibition of EGFR or β1 by blocking antibodies and other inhibitors induces downregulation of EGFR expression and phosphorylation in parallel with the reversion of breast tumor cells to a phenotypically normal morphology. This cross-modulation occurs only in a three-dimensional assay of laminin-rich gels, suggesting that EGFR and β1 cross-talk requires a malleable BM [58]. β1 induces the assembly of macromolecular complexes containing c-Src and Crk-associated substrate p130Cas, and leads to phosphorylation of specific EGFR tyrosine residues [59]. β1 is critical not only in mediating the activation but also in promoting EGFR1, ErbB2 and Met recycling, increasing the signaling to induce metastasis [60]. Reciprocally, EGFR signaling promotes the invasiveness of some breast cancer cells via integrin recycling [61].

Amplification or overexpression of ErbB2 is associated with poor survival. In the MMTV/ErbB2 mouse model, β1-deficient tumor metastasis to the lung was delayed and was correlated with downregulation of tyrosine phosphorylation of Src, p130Cas and paxillin, all integrin-coupled signaling partners [40]. The integration of signaling between ErbB2 and β1 has relevant clinical implications. Notably, β1 overexpression was identified as an independent negative prognostic marker in ErbB2-positive breast cancer patients treated with trastuzumab, and β1-activated pathways such as PI3K/Akt or Erk circumvented the antiproliferative activity of trastuzumab in breast cancer cell lines [10]. Lapatinib resistance has been reported to be mediated by β1-induced FAK and Src activity [11]. Of clinical relevance, blocking β1 with the blocking antibody AIIB2 restores the growth inhibitory effects of both trastuzumab [10] and lapatinib individually or of the lapatinib and trastuzumab combination [11]. β1 is also involved in tamoxifen resistance, and fibroblasts, derived from an estrogen-dependent, spontaneous mammary tumor, produce soluble factors inducing tamoxifen resistance in the epithelial cells through activation of EGFR, PI3K/AKT and β1. Binding of FN to β1 confers resistance, and treatment with the anti-β1 blocking antibody restores cell death in response to tamoxifen [9].

Unlike β1, which exerts its function by recruiting signaling adaptor proteins, β4 has unique signaling properties. These properties derive from its long cytoplasmic tail, able to activate the PI3K pathway with tyrosine and serine phosphorylation sites, which combine with – and enhance – the signaling of ErbB2, EGFR and Met. Keeping in mind the cross-talk between β4 and RTKs, this integrin has been involved in promoting resistance to anti-epidermal growth factor family receptor targeted therapy. RTK activation induces hemidesmosome disassembly and β4 phosphorylation [62], which in turn activate different signaling pathways that control transcriptional regulators and amplify the RTK signaling in MMTV-Neu/β4 mice [44]. However, deletion of the β4 signaling domain improves the efficacy of anti-RTK therapy [44].

In humans, β4 contributes to tamoxifen resistance through regulation of ErbB3 expression in breast cancer cells. β4 and ErbB3 expression correlates in p-Akt-positive, ERβ1-negative breast tumor samples [14]. Finally, ligand-activated β4 is directly involved in tissue polarity and in a program leading to resistance to apoptosis-inducing drugs [28], again indicating the critical role of tissue architecture as a crucial indicator of tumor behavior and sensitivity/resistance to anti-apoptotic therapies [28].

Radiation therapy is an effective primary modality in the treatment of breast cancer, even though tumor radio-resistance remains a significant clinical problem [63]. In addition to DNA damage, radiation induces several changes in cell–cell and cell–ECM interactions, and thereby modulates the tumor microenvironment [13]. Much of the data available on the role of β1 in resistance to therapy concern its overexpression in radio-resistant tumors. Radiation induces β1 robustly in several solid malignancies including breast cancer [13],[64]-[66], and specifically upregulates α5β1 and its primary ligand, FN [13]. Radiation-induced β1 expression is associated with activation and increased binding of nuclear factor-κB to the β1 promoter region, resulting in increased transcriptional activity [65]. Addition of β1 inhibitory antibodies enhances the growth-inhibitory efficacy of radiotherapy in three-dimensional culture models of breast cancer cells and in mice bearing MCF-7 xenografts. This inhibition was associated with a downregulation of radiation-induced integrins and downstream Akt activity [64].

DCIS lesions, despite being separated from the stromal tissues by BM, show stromal remodeling of ECM, and may progress to invasive cancer after radiation therapy. Recently, a subgroup of patients who had local recurrence after radiation treatment was shown to have overexpression of β1 integrin and a high expression of p-AKT in their DCIS lesions [7]. AKT-overexpressing MCF10A cells grown in three-dimensional laminin-rich ECM, as well as injected into the mouse mammary duct, resemble DCIS-like lesions. When treated with ionizing radiation, luminal cells of organotypic DCIS-like lesions preferentially undergo apoptosis, whereas surviving cells show upregulated FN and α5β1 integrin, increased MMP9 activity, loss of E-cadherin, nuclear localization of nuclear factor-κB and invasive ability [7].

In summary, signaling through β1 and β4 has been shown to be responsible for resistance to different therapeutic strategies including radiotherapy, chemotherapy, hormone therapy and targeted therapies. Targeting these signaling pathways could thus probably also circumvent therapy resistance in early-stage cancer patients.

Cell survival and resistance to apoptosis require β1-mediated signals derived from specific ECM components, and BM provides a microenvironment that allows formation of polar structures [28], quiescence [27],[67] and resistance to apoptosis [68].

When metastatic cells cross the BM of the microvasculature, they become dormant on mature BM surrounding endothelial cells. Close to the sprouting new vasculature, however, dormant cells are awakened and form tumors that will metastasize [69]. The translation of these recent findings into clinical practice holds much promise with a new scenario in the designing of effective therapies, in particular those based on radiation or other chemotherapeutic agents combined with reduced doses of radiation [64].

## Conclusions

β1 and β4 integrins, although structurally different, participate in mammary gland development and maintain the integrity of mammary architecture; they contribute to cell and tissue polarity and to the function of the mammary gland. Deregulation of these pathways, from ECM to transcription factors, alters cell adhesion and the integrity of the tissues and organs and contributes to invasiveness, breast cancer progression and therapy resistance.

The relevance of integrins in tumor progression warrants the development of effective antagonists for clinical use and is an emerging therapeutic field. To our knowledge, however, there are currently no clinical trials with β4 inhibitors. To inhibit β1, an α5β1 inhibiting peptide (ATN-161) has been employed in phase II trials, also in combination with chemotherapy [70], and a monoclonal anti-β1 antibody is under development. We believe the efforts being made in the field to identify integrin-related pathways and specific inhibitors that influence tumors and their microenvironments may lead to more efficacious combined treatments.

## Authors’ original submitted files for images

Below are the links to the authors’ original submitted files for images. Authors’ original file for figure 1

## Abbreviations

BM: Basement membrane
DCIS: Ductal carcinoma *in situ*
ECM: Extracellular matrix
EGFR: Epidermal growth factor receptor
EMT: Epithelial–mesenchymal transition
FAK: Focal adhesion kinase
FN: Fibronectin
ILK: Integrin-linked kinase
MAPK: Mitogen-activated protein kinase
MMP: Matrix metalloproteinase
MMTV: Mouse mammary tumor virus
PI3K: Phosphatidylinositol-3-kinase
RTK: Receptor tyrosine kinase
STAT: Signal transducer and activator of transcription
TGFβ: Transforming growth factor beta
