# Supplementary figures and images for: β1 and β4 integrins: from breast development to clinical practice
Source: Breast Cancer Res. 2014 Oct 30;16:459. doi: 10.1186/s13058-014-0459-x (PMC4384274; doi:10.1186/s13058-014-0459-x)

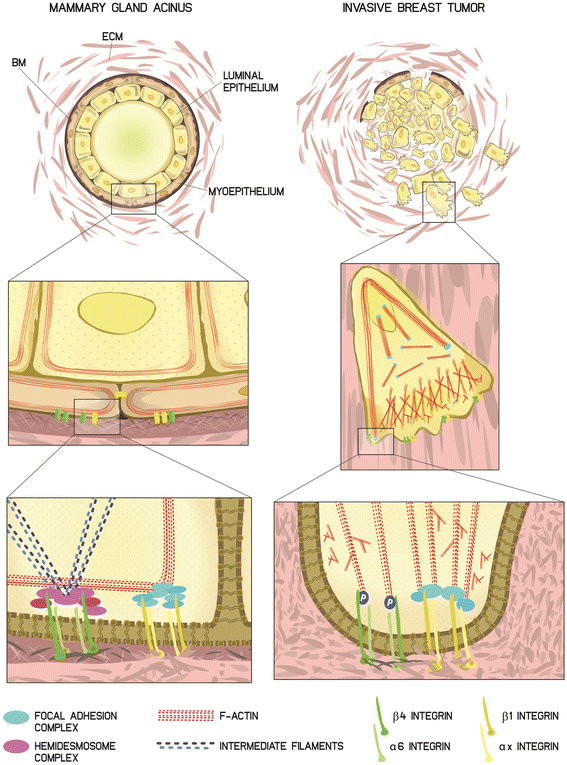

Supplement: Supplementary file 1 — Authors’ original file for figure 1 [file 13058_2014_459_MOESM1_ESM.gif]
